# Antimicrobial resistance, genetic diversity and virulence associated factors of *Campylobacter* spp. isolated from poultry meat in Algeria

**DOI:** 10.3389/fvets.2026.1819023

**Published:** 2026-05-05

**Authors:** Radia Bouhamed, Taha-Mossadak Hamdi, Cemil Kurekci, Herbert Tomaso, Heinrich Neubauer, Hosny El-Adawy

**Affiliations:** 1 Laboratory of Research Food Hygiene and Quality Insurance System (HASAQ), Higher National Veterinary School Rabie BOUCHAMA (ENSV), Algiers, Algeria; 2 Faculty of Veterinary Medicine, Department of Microbiology, Dokuz Eylul University, İzmir, Türkiye; 3 Institute of Bacterial Infections and Zoonoses, Friedrich-Loeffler-Institut, Jena, Germany; 4 Faculty of Veterinary Medicine, Kafrelsheikh University, Kafr El-Sheikh, Egypt

**Keywords:** Algeria, antimicrobial resistance, *fla*A genotyping, poultry meat, thermotolerant *Campylobacter*, virulence genes

## Abstract

**Background:**

*Campylobacter* is the most common cause of bacterial food infections worldwide. In Algeria, data regarding the epidemiology, antimicrobial resistance and virulence of *Campylobacter* remain limited. This study aimed to investigate the antimicrobial resistance, genotyping and virulence associated mechanism of *Campylobacter* isolated from slaughterhouses in Algeria.

**Methods:**

A total of 133 pooled poultry samples were collected from five slaughterhouses in Algiers. *Campylobacter* spp. was differentiated and the antimicrobial susceptibility testing against 12 antimicrobial agents was assessed using Kirby-Bauer-Method. The *tet*O gene and mutations in the *gyr*A gene associated with tetracyclines and fluoroquinolone resistance were investigated, respectively. The potential associated genes were screened. The *fla*A typing for isolated *Campylobacter* was performed using PCR-RFLP.

**Results:**

*Campylobacter* spp. was detected in 28 (21.05%) poultry samples. The prevalence was significantly higher in caecal samples (61.11%) than in neck skin samples (14.78%). Among 28 isolates, 19 (67.86%), 8 (28.57%) and 1 (3.57%) were identified as *Campylobacter coli*, *Campylobacter jejuni* and *Campylobacter lari*, respectively. High levels of resistance to fluoroquinolones (92.86%), nalidixic acid (96.43%) and tetracycline (85.71%) were observed. Multidrug resistance to three or more classes of antimicrobial agents was ranged between 7.14 and 28.57%. The *tet*O gene was amplified in 20 (83.3%) phenotypic tetracycline resistant isolates while mutations in the *gyr*A gene associated with fluoroquinolone resistance were detected in 84.62% (22/26) of isolates. *Campylobacter* spp. was differentiated into 16 genotypes and two isolates from human were belonged to two different *fla*A genotypes. All 19 *Campylobacter coli* isolates carried *cad*F and *cdt*A, while *rac*R, *dna*J, *cdt*B and *cdt*C were detected in 36.84, 36.84, 84.21 and 52.63%, respectively. In turkeys, 22.22% of *Campylobacter coli* and 100% of *Campylobacter jejuni* harbored all virulence associated genes.

**Conclusion:**

These findings emphasize the importance of integrated surveillance of *Campylobacter* across animal and human sectors to mitigate public health risks.

## 1 Introduction

Thermotolerant *Campylobacter* (*C*.), primarily *C. jejuni* and *C. coli*, are recognized as the leading bacterial cause of human gastroenteritis worldwide and represent a major public health concern (1). These pathogens are considered priority foodborne bacteria by international health authorities due to their high incidence, zoonotic nature and increasing antimicrobial resistance.

Poultry constitute the main reservoir of thermotolerant *Campylobacter* and plays a central role in the epidemiology of Campylobacteriosis (2). Human infection is predominantly associated with the handling and consumption of contaminated poultry products (3). Consequently, contamination at any stage of the poultry production and processing chain may directly impact human health.

The Algerian poultry industry produces annually on average 340.000 tons of white meat and more than 4.8 billion eggs (4). In Algeria, poultry meat, particularly broiler chicken and turkey, represents the most consumed animal protein source due to its affordability and nutritional value (5). Turkey meat is especially appreciated for its low-fat content, making it suitable for consumers with dietary or cholesterol-related constraints (6). Poultry meat consumption per capita reached 9.88 kg in 2023 in Algeria, according to Faostat (7).

The use of a direct isolation method inspired by modified ISO 10272-1 (2017) could represent a good alternative to the indirect isolation method in developing countries, as it would increase the detection rate of *Campylobacter* spp. due to the decrease in detection time, costs and contaminant rates (8). *Campylobacter* spp. presents a significant risk for transmission from infected animals or animal-derived products to humans, animals and the environment. Recent research highlighted poultry products as a primary reservoir, with domestic poultry implicated in various regions worldwide.

Africa has been identified as one of the endemic regions of campylobacteriosis. However, research attention is not geared toward *Campylobacter* source tracing like other enteropathogens, probably because of inadequate standard laboratory facilities and the fastidious nature of the organism. It was noted that the common and emerging species of *Campylobacter* have been documented in Africa and the major transmission pathways to humans are international travel and consumption of contaminated poultry (9).

The results of previous study in Algeria showed a high prevalence of thermotolerant *Campylobacter* with multidrug resistance profiles in poultry farms and slaughterhouses of Algiers (10). Potential sources of contamination of *Campylobacter* species were noticed in farms and slaughterhouses located in the middle area of Algeria. The most tested strains exhibited resistance to ciprofloxacin (75%) and erythromycin (25%) (11).

Despite the importance of poultry production, data on the prevalence, antimicrobial resistance and virulence of thermotolerant *Campylobacter* in Algeria remain scarce. In foods of animal origin, these bacteria may enter a viable but non-culturable (VBNC) state under environmental stress, enabling survival and persistence along the food chain (12). Although Campylobacteriosis is often self-limiting, severe infections require antimicrobial treatment, particularly in vulnerable populations (13).

*Campylobacter* is genetically highly diverse and undergoes frequent intraspecific recombination. Turkeys have been identified as an important reservoir for *C. jejuni* which is of public health significance (14). The emergence of resistance to critically important antimicrobials, notably fluoroquinolones and macrolides (15, 16), has further increased the public health relevance of *Campylobacter* (17, 18). This antimicrobial resistance threatens treatment efficacy and highlights the need for integrated surveillance at the human–animal–environment interface (19, 20).

Therefore, the present study aimed to investigate thermotolerant *Campylobacter* isolated from poultry in Algeria by assessing prevalence, antimicrobial resistance patterns, resistance mechanisms, and potential virulence gene profiles epidemiological links within their pathogenicity.

## 2 Materials and methods

### 2.1 Sampling strategy and sample collection

The sampling was conducted over a period extending in one year from January to November. Samples were collected across multiple seasons, with a higher representation in winter (13 samples), followed by autumn (6 samples) and spring (3 samples). Furthermore, a total of 7 different cities contributed to the sampled batches. The samples were collected at the slaughterhouses and not on farm site, that’s why we cannot neither determine whether the farms located in the same city have the same origin nor provide the number of farms contributing to the batches. A total of 133 pooled samples resulting from 460 individual collections (115 neck skin samples and 18 caecal samples), including broiler chickens and turkeys, were aseptically and randomly collected from 23 poultry batches in five poultry slaughterhouses in the Algiers region, Algeria.

The caecal samples could not be collected from all 23 batches but only from 18 batches. In five batches (batches 1, 2, 3, 4 and 8), the slaughterhouse staff had discarded the ceca before my arrival. From the 23 visited poultry batches, 23 neck skins samples and 18 cecal samples were collected.

Fifteen carcasses were sampled per batch and approximately 10 g of neck skin were taken from each carcass. Neck skins from three carcasses of the same batch were pooled to obtain five composite samples per batch (21). Caecal samples were collected immediately after evisceration. Five caeca from five birds of the same batch were pooled to form a single sample. All samples were placed in sterile containers, transported in insulated boxes under refrigerated conditions, and processed within 2–4 h of collection (22).

### 2.2 Isolation and identification of *Campylobacter*

Caecal contents were aseptically removed and pooled. One gram of pooled caecal content was homogenized in 9 mL of sterile 0.9% physiological saline. For neck skin samples, 10 g of pooled skin were added to Bolton selective enrichment broth at a 1:10 ratio and homogenized in sterile stomacher bags. Isolation of thermotolerant *Campylobacter* was performed according to ISO 10272:2017 (21). Samples were streaked onto modified Charcoal Cefoperazone Deoxycholate Agar (mCCDA) (Oxoid, Wesel, Germany) and incubated at 42 °C for 48 h under microaerophilic conditions (5% O_2_, 10% CO_2_, 85% N_2_) (Anoxomat®, Advanced Instrument, USA). Presumptive colonies were sub-cultured on Columbia blood agar (Oxoid, Wesel, Germany) for purification.

Identification was based on colony morphology, Gram staining, oxidase and catalase tests, growth at different temperatures and biochemical characteristics, including susceptibility to nalidixic acid and cephalothin. Confirmation was achieved using latex agglutination tests, API Campy galleries (bioMérieux, France) and confirmed by PCR assays (23–25).

### 2.3 Antimicrobial susceptibility testing

Antimicrobial susceptibility test of isolated *Campylobacter* was performed against 12 antimicrobial agents using the Kirby–Bauer test according to EUCAST guidelines (26).

Zones diameters indicating resistance to erythromycin, tetracycline and ciprofloxacin were analyzed according to the EUCAST breakpoints for *Campylobacter* (26). While different reference interpretive standards according to CLSI breakpoints for *Enterobacteriaceae* were used for a tentative classification of the isolates as susceptible or resistant against Amoxicillin/Clavulanic acid (AMC), Ampicillin (AM), Cephotaxime (CTX) according to the EUCAST breakpoints (26). For Streptomycin (S), Gentamicin (GM), Kanamycin (K), Tobramycin (TM), Nalidixic Acid (NA) and Chloramphenicol (C) (27).

*Campylobacter jejuni* DSM 4688 (ATCC 33560) and *C. coli* DSM 4689 (ATCC 33559) (Deutsche Sammlung von Mikroorganismen und Zellkulturen GmbH, Braunschweig, Germany) were used as reference strains for quality control in each batch of the antimicrobial susceptibility tests.

### 2.4 DNA extraction

Genomic DNA was extracted from pure cultures using a thermal lysis method. Briefly, *Campylobacter* spp. isolates were preserved on Columbia agar (Oxoid, Wesel, Germany). After incubation at 42 °C for 48 h under microaerophilic conditions, isolated *Campylobacter* colonies were harvested from pure cultures for DNA extraction. Colonies were transferred into sterile Eppendorf tubes containing 400 μL of sterile nuclease-free water (DNA- and RNA-free; Thermo Scientific™, HyClone® HyPure™). The suspensions were incubated at 100 °C for 10 min using a digital dry bath (Benchmark™, MyBlock™). Following lysis, samples were centrifuged at 10,000 rpm for 5 min using a microcentrifuge (Beckman Coulter®, Microfuge® 16). The resulting supernatants were transferred into sterile 1.5 mL microtubes and stored at −20 °C until PCR analysis.

### 2.5 Molecular identification and genotyping

*Campylobacter* spp. was identified and differentiated into *C. jejuni*, *C. coli* and *C. lari* using multiplex PCR (25) (Table 1). The PCR was performed in a 50-μL reaction mixture containing 5.0 μL of 10 × Taq reaction buffer complete (Jena Bioscience GmbH, Jena, Germany), 2.0 μL of dNTP mix (2 mM each; Carl Roth GmbH, Karlsruhe, Germany), 2.0 μL of each primer (Jena Bioscience GmbH; Table 2) and 0.2 μL of Taq Pol thermostable DNA polymerase (Jena Bioscience GmbH). Amplification reactions were carried out in Eppendorf Thermoblock cycler (Eppendorf, Germany) using the following program: one cycle of 1 min at 96 °C followed by 35 cycles each consisting of 60 s at 95 °C, 90 s at 59 °C and 60 s at 72 °C. The PCR was terminated by a final extension step of 5 min at 72 °C. Amplification generated 650, 323, and 126 bp DNA fragments specific for the genus *Campylobacter*, *C. jejuni* and *C. coli*, respectively. The corresponding amplicon for *C. lari* was 522 bp. For analysis, 20 μL of PCR products were subjected to electrophoresis in a 1.5% agarose gel for 1 h, stained with ethidium bromide (0.5 μg/mL), and visualized under UV light. Results were documented using BioImage system GeneGenius (Syngene, Synoptics Ltd., Cambridge, UK). Reference strains *C. jejuni* DSM 4688 (ATCC 33560), *C. coli* DSM 4689 (ATCC 33559) and *C. lari* DSM 11375 (ATCC 352219) (Deutsche Sammlung von Mikroorganismen und Zellkulturen GmbH, Braunschweig, Germany) were used as positive controls.

**Table 1:** Primer sequences and expected amplicon sizes used in this study.

Target	Primer	Size (bp)	Sequences (5′-3′)	Reference
*Campylobacter* spp.	23SF	650	TATACCGGTAAGGAGTGCTGGAG	(25)
	23SR		ATCAATTAACCTTCGAGCACCG	
*Campylobacter jejuni*	CjF	323	ACTTCTTTATTGCTTGCTGC	
	CjR		GCCACAACAAGTAAAGAAGC	
*Campylobacter coli*	CcF	126	GTAAAACCAAAGCTTATCGTG	
	CcR		TCCAGCAATGTGTGCAATG	
*Campylobacter lari*	CL 1155 R	522	ATT TAG AGT GCT CAC CCG AAG	(24)
	CL 632-rv		GGG AAA CTG GTA ATC TAG AGT GG	
*tetO*	tet1	559	GGCGTTTTGTTTATGTGCG	(28)
	tet2		ATGGACAACCCGACAGAAGC	
*gyrA*	GzgyrA5	673	ATTTTTAGCAAAGATTCTGAT	(29)
	GzgyrA6		CCATAAATTATTCCACCTGT	
*cdt*A	GNW	165	GGAAATTGGATTTGGGGCTATACT	(30)
	IVH		ATCACAAGGATAATGGACAAT	
*cdt*B	VAT2	495	GTTAAAATCCCCTGCTATCAACCA	
	WMI-R		GTTGGCACTTGGAATTTGCAAGGC	
*cdt*C	WMI-F	555	TGGATGATAGCAGGGGATTTTAAC	
	LPF-X		TTGCACATAACCAAAAGGAAG	
*cad*F	F2B	400	TTGAGGGTAATTTAGATATG	(32)
	R1B		CTAATACCTAAAGTTGAAAC	
*rac*R	25	584	GATGATCCTGACTTTG	(31)
	593		TCTCCTATTTTTACCC	
*dna*J	299	720	AAGGCTTTGGCTCATC	
	1003		CTTTTTGTTCATCGTT	

**Table 2:** Prevalence of thermotolerant *Campylobacter* isolated from 133 pooled samples.

Sample source	Samples number	Isolates *n* (%)	*Campylobacter coli n* (%)	*Campylobacter jejuni n* (%)	*Campylobacter lari n* (%)	95% confidence interval
Neck skin	115	17 (14.78)	12 (70.59)^*^	5 (29.41)^*^	0^*^	[8.3–21.3%]
Broilers	70	10 (14.29)^*^	7 (70)	3 (30)	0	[7.07–24.71%]
Turkeys	45	7 (15.56)^*^	5 (71.43)	2 (28.57)	0	[6.49–29.46%]
Caecal content	18	11 (61.11)	7 (63.64)^*^	3 (27.27)^*^	1 (9.09)^*^	[35.75–82.70%]
Broilers	12	6 (50)^*^	3 (50)	2 (33.33)	1 (16.67)	[21.09%–78.91%]
Turkeys	6	5 (83.33)^*^	4 (80)	1 (20)	0	[35.88%–99.58%]
Total	133	28 (21.05)	19 (67.86)	8 (28.57)	1 (3.57)	[14.1–28%]

^*^p > 0.05.

### 2.6 Molecular detection of resistance and potential virulence associated mechanisms

The antimicrobial resistant genes (*tet*O) associated with phenotypic tetracycline resistance (28) and mutations in the *gyr*A gene were associated with fluoroquinolone resistance (29) were identified by PCR.

The potential virulence-associated genes (*cdt*A, *cdt*B, *cdt*C, *cad*F, *rac*R and *dna*J) were amplified using forward and reverse primers in Table 1 and PCR reaction was performed according to the previously published protocol (30–32).

### 2.7 *fla*A-RFLP assays

For *fla*A-RFLP analysis, extracted DNA was amplified as described previously (14, 33). Out of 28 *Campylobacter* isolated in this study, *fla*A gene was only amplified in 22 isolates. Two *C. jejuni* isolated from humans suffering from campylobacteriosis were included in the analysis. Amplification conditions were: initial denaturation for 60 s at 94 °C followed by 35 cycles each consisting of 15 s at 94 °C, 60 s at 45 °C, 120 s at 72 °C and a final extension step of 300 s at 72 °C. The *fla*A amplicon was digested for 18 h at 37 °C with *Sau3*AI (Jena Bioscience GmbH) and *Dde*I (Roche Diagnostics GmbH) enzymes using the incubation buffer as recommended by the manufacturers. The DNA segments were separated using 2.5% agarose gels (Starlab GmbH, Hamburg, Germany) in TBE buffer at 200 V for 1 h, stained with ethidium bromide and visualized under UV light. Documentation was done using a Bio Imaging System (Syngene, Cambridge, UK). TIF images of the restriction profiles for *fla*A-RFLP were incorporated for analysis into BioNumerics V. 7.1 (Applied Maths, Austin, TX, USA). Pair comparisons and cluster analysis were made using the Dice correlation coefficient and the unweighted pair group mathematical average (UPGMA) clustering algorithm. The optimization and position tolerance for band analysis were set at 4%, and a cut-off of 90% was used for the determination of the different restriction patterns for *fla*A-RFLP.

### 2.8 Statistical analysis

The results were analyzed using AnaStats software. The tests performed included the calculation of 95% confidence intervals (95% CI), as well as the Chi-square (χ^2^) test, Fisher’s exact test and G test (log-likelihood ratio test). Differences were considered statistically significant when the probability (*p*) was less than or equal to the significance level *α* (*p* ≤ 0.05).

## 3 Results

### 3.1 Prevalence of thermotolerant *Campylobacter*

Out of 133 investigated poultry samples, thermotolerant *Campylobacter* was detected and confirmed by PCR in 28 (21.05%) samples. The prevalence was significantly higher in caecal samples (61.11%; 11/18) than in neck skin samples (14.78%; 17/115). Among 28 isolates, 19 (67.86%), 8 (28.57%) and 1 (3.57%) were identified as *C. coli*, *C. jejuni* and *C. lari*, respectively using PCR (Table 2).

The epidemiological distributions of the isolated *Campylobacter* according to the sample type and locations of slaughter houses were demonstrated in Table 3.

**Table 3:** Epidemiological distribution of isolated *Campylobacter* from slaughter houses in Algeria.

Source		*Campylobacter* species	Location	Number of birds	Age	Sex
Turkey	Neck skin	*Campylobacter coli*	Tizi Ouzou	20	5 months	♂
		*Campylobacter coli*	Issers	40	5 months	♂ + ♀
		*Campylobacter coli*				
		*Campylobacter coli*	Tizi Ouzou	30	5 months	♂
		*Campylobacter coli*				
		*Campylobacter jejuni*	Sétif	100	4 months	♂
		*Campylobacter jejuni*				
	Caecal content	*Campylobacter coli*	Tizi Ouzou	30	5 months	♂
		*Campylobacter coli*	Tizi Ouzou	30	5 months	♂
		*Campylobacter jejuni*	Sétif	100	4 months	♂
		*Campylobacter coli*	Tablat	30	5 months	♂
		*Campylobacter coli*	Sétif	100	4 months	♂
Broiler	Neck skin	*Campylobacter jejuni*	Médéa	600	60 days	♂
		*Campylobacter coli*				
		*Campylobacter jejuni*	Médéa	500	60 days	♂ + ♀
		*Campylobacter coli*	Sétif	1,400	44 days	♂ + ♀
		*Campylobacter coli*	Boumerdes	700	60 days	♂ + ♀
		*Campylobacter coli*				
		*Campylobacter coli*	Boumerdes	700	60 days	♂ + ♀
		*Campylobacter coli*				
		*Campylobacter jejuni*				
		*Campylobacter coli*				
	Caecal content	*Campylobacter coli*	Médéa	600	60 days	♂ + ♀
		*Campylobacter lari*	Boumerdes	250	55 days	♂ + ♀
		*Campylobacter coli*	Tablat	350	60 days	♂ + ♀
		*Campylobacter jejuni*	Sétif	1,300	60 days	♂ + ♀
		*Campylobacter coli*	Bejaia	700	58 days	♂ + ♀
		*Campylobacter jejuni*	Bejaia	700	60 days	♂ + ♀

### 3.2 Antimicrobial resistance profiles

A high frequency of resistance to fluoroquinolones and tetracycline was observed among isolated *Campylobacter*. Resistance to macrolides was less frequent but was detected in some isolates (Table 4).

**Table 4:** Breakpoints and Antimicrobial susceptibility patterns of *Campylobacter* spp. identified by the disk diffusion methods using disk diffusion methods (26, 27).

Antimicrobial agents	Disk content	Breakpoints (mm)		Resistance n (%)	95% confidence interval	References
		S^*^	R^*^			
Ampicillin (AM)	10 μg	≥ 14	< 14	21 (75%)	[55.13–89.31%]	(26)
Amoxicillin/Clavulanic acid (AMC)	20/10 μg	≥ 50	< 19	12 (42.86%)	[24.46–62.82%]	(26)
Cefotaxime (CTX)	5 μg	≥ 20	< 17	14 (50%)	[30.65–89.35%]	(26)
Streptomycin (S)	10 μg	≥ 15	< 11	10 (35.71%)	[18.64–55.93%]	(27)
Gentamicin (GM)	10 μg	≥ 15	< 12	0	[0–12.34%]	(27)
Kanamycin (K)	30 μg	≥ 18	< 13	14 (50%)	[30.65–89.35%]	(27)
Tobramycin (TM)	10 μg	≥ 15	< 12	15 (53.57%)	[33.87–72.49%]	(27)
Erythromycin (E)	15 μg	≥ 20 (18)	< 20 (18)	15 (53.57%)	[33.87–72.49%]	(26)
Nalidixic acid (NA)	30 μg	≥ 20	< 15	27 (96.43%)	[81.64–99.91%]	(27)
Ciprofloxacin (CIP)	5 μg	≥ 50	< 26	26 (92.86%)	[76.50–99.12%]	(26)
Tetracycline (TE)	30 μg	≥ 30	< 30	24 (85.71%)	[67.33–95.97%]	(26)
Chloramphenicol (C)	30 μg	≥ 18	< 12	1 (3.57%)	[0.09–18.35%]	(27)

^*^S, susceptible; R, resistant.

The highest resistance rates were observed for ampicillin (75%, 21/28; 95% CI: 55.13–89.31%), tetracycline (85.71%, 24/28; 95% CI: 67.33–95.97%), ciprofloxacin (92.86%, 26/28; 95% CI: 76.50–99.12%) and nalidixic acid (96.43%, 27/28; 95% CI: 81.64–99.91%) (*p* > 0.05). Approximately 50% of isolates were resistant to amoxicillin/clavulanic acid (42.86%, 12/28; 95% CI: 24.46–62.82%), cefotaxime (50%, 14/28; 95% CI: 30.65–89.35%), kanamycin (50%, 14/28; 95% CI: 30.65–89.35%), tobramycin (53.57%, 15/28; 95% CI: 33.87–72.49%) and erythromycin (53.57%, 15/28; 95% CI: 33.87–72.49%). Lower resistance rates were recorded for streptomycin (35.71%, 10/28; 95% CI: 18.64–55.93%) (*p* > 0.05). Chloramphenicol resistance was rare (3.57%, 1/28; 95% CI: 0.09–18.35%) and no resistance to gentamicin was observed (0%, 0/28; 95% CI: 0–12.34%). Additionally, 92.86% (26/28) of *Campylobacter* isolates exhibited cross-resistance between nalidixic acid and ciprofloxacin, while one isolate was resistant to nalidixic acid but sensitive to ciprofloxacin (3.57%, 1/28).

Multidrug resistance to three or more classes of antimicrobial agents was ranged between 7.14 and 28.57% of *Campylobacter* isolates, as shown in Table 5.

**Table 5:** Multi-drug-resistance (MDR) rates of thermotolerant *Campylobacter* against different antimicrobial classes.

MDR profiles	No. (%)
Resistance against 3 classes	2 (7.14)
Tetracyclines; Aminoglycosides; Fluoroquinolones	1 (3.57)
Fluoroquinolones; Penicillin; Macrolides	1 (3.57)
Resistance against 4 classes	3 (10.7)
β-Lactames; Tetracyclines; Aminoglycosides; Fluoroquinolones	1 (3.57)
β-Lactames; Tetracyclines; Fluoroquinolones; Penicillin	1 (3.57)
Tetracyclines; Aminoglycosides; Fluoroquinolones; Macrolides	1 (3.57)
Resistance against 5 classes	8 (28.57)
Tetracyclines; Aminoglycosides; Fluoroquinolones; Penicillin; Macrolides	1 (3.57)
Cephalosporins; Tetracyclines; Fluoroquinolones; Penicillin; Macrolides	1 (3.57)
Tetracyclines; Fluoroquinolones; Penicillin; Cephalosporins; Aminoglycosides	1 (3.57)
β-Lactames; Tetracyclines; Fluoroquinolones; Penicillin; Aminoglycosides	1 (3.57)
β-Lactames; Tetracyclines; Fluoroquinolones; Penicillin; Aminoglycosides	1 (3.57)
Tetracyclines; Fluoroquinolones; Penicillin; Aminoglycosides; Cephalosporins	1 (3.57)
Tetracyclines; Fluoroquinolones; Penicillin; Aminoglycosides; Macrolides	2 (7.14)
Resistance against 6 classes	8 (28.57)
β-Lactames; Tetracyclines; Fluoroquinolones; Penicillin; Aminoglycosides; Cephalosporins	4 (14.3)
β-Lactames; Tetracyclines; Fluoroquinolones; Penicillin; Aminoglycosides; Macrolides	1 (3.57)
Tetracyclines; Fluoroquinolones; Penicillin; Aminoglycosides; Macrolides; Cephalosporins	1 (3.57)
β-Lactames; Tetracyclines; Fluoroquinolones; Aminoglycosides; Macrolides; Cephalosporins	1 (3.57)
Tetracyclines; Fluoroquinolones; Penicillin; Aminoglycosides; Macrolides; Cephalosporins	1 (3.57)
Resistance against 7 classes	4 (14.29)
β-Lactames; Tetracyclines; Fluoroquinolones; Penicillin; Aminoglycosides; Macrolides; Cephalosporins	3 (10.7)
Tetracyclines; Fluoroquinolones; Penicillin; Aminoglycosides; Macrolides; Cephalosporins; Phenicol	1 (3.57)

### 3.3 Molecular detection of resistance and virulence associated genes

Out of 24 phenotypically tetracycline resistant *Campylobacter*, *tet*O gene associated with tetracycline-resistant was amplified in 20 (83.3%) isolates while mutations in the *gyr*A gene associated with fluoroquinolone resistance were detected in 84.62% (22/26) of isolates.

Three colonization genes (*cad*F, *rac*R, *dna*J) and three pathogenicity genes (*cdt*A, *cdt*B, *cdt*C) were screened in all *Campylobacter* spp. isolates (Table 6). All 19 *C. coli* isolates carried *cad*F and *cdt*A, while *rac*R, *dna*J, *cdt*B and *cdt*C were detected in 36.84% (7/19), 36.84% (7/19), 84.21% (16/19) and 52.63% (10/19), respectively. A three-gene (*cad*F-*rac*R-*dna*J) profile was present in *C. coli* from broilers (30%, 3/10; 95% CI: 6.67–65.25%) and turkeys (22.22%, 2/9; 95% CI: 2.81–60.01%).

**Table 6:** Prevalence of colonization and pathogenicity associated genes for *Campylobacter* spp.

Gene	*Campylobacter coli* (*n* = 19)			95% confidence interval	*Campylobacter jejuni* (*n* = 8)			95% confidence interval	*Campylobacter lari* (*n* = 1)
	Broiler (*n* = 10)	Turkey (*n* = 9)	Total *n* (%)		Broiler (*n* = 5)	Turkey (*n* = 3)	Total *n* (%)		Broiler *n* (%)
*cad*F	10 (100)	9 (100)	19 (100)	82.3–100%	4 (80)	3 (100)	7 (87.5)	47.35–99.68%	0
*rac*R	4 (40)	3 (33.33)	7 (36.8)	16.29–61.64%	3 (60)	3 (100)	6 (75)	34.91–96.81%	1 (100)
*dna*J	3 (30)	4 (44.44)	7 (36.8)	16.29–61.64%	4 (80)	3 (100)	7 (87.5)	47.35–99.68%	0
*cad*F-*rac*R	1 (10.0)	1 (11.11)	2 (10.5)	0–24.3%	0	0	0	0–32.0%	0
*cad*F-*dna*J	0	2 (22.22)	2 (10.5)	0–24.3%	1 (20)	0	1 (12.5)	0.32–52.65%	0
*cad*F-*rac*R-*dna*J	3 (30)	2 (22.22)	5 (26.3)	6.5–46.1%	3 (60)	3 (100)	6 (75)	34.91–96.81%	0
*cdt*A	10 (100)	9 (100)	19 (100)	82.3–100%	4 (80)	3 (100)	7 (87.5)	47.35–99.68%	0
*cdt*B	9 (90)	7 (77.78)	16 (84.2)	60.42–96.62%	4 (80)	3 (100)	7 (87.5)	47.35–99.68%	0
*cdt*C	3 (30)	7 (77.78)	10 (52.6)	28.86–75.55%	2 (40)	3 (100)	5 (62.5)	24.49–91.48%	0
*cdt*A-*cdt*B	1 (10)	0	1 (5.3)	10.7–52.5%	0	0	0	0–55.0%	0
*cdt*A*-cdt*B*-cdt*C	3 (30)	7 (77.78)	10 (52.6)	28.86–75.55%	5 (62.5)	3 (100)	8 (100)	24.49–91.48%	0
*cad*F*-rac*R*-dna*J*-cdt*A*-cdt*B*-cdt*C	0	2 (22.22)	2 (10.5)	0–24.3%	2 (40)	3 (100)	5 (62.5)	24.49–91.48%	0

Most *C. coli* isolated from poultry carried at least one pathogenicity gene (15.79%, 3/19; 95% CI: 3.38–39.58%). Most frequently observed genes in *C. jejuni* isolates were *cad*F, *dna*J, *cdt*A and *cdt*B (87.5%, 7/8; 95% CI: 47.35–99.68%), while *rac*R and *cdt*C were present in 75% (6/8; 95% CI: 34.91–96.81%) and 62.5% (5/8), respectively.

The majority of *C. jejuni* (75%, 6/8; 95% CI: 34.91–96.81%) carried all three colonization genes *cad*F-*rac*R-*dna*J and represented as broilers (60%, 3/5; 95% CI: 14.66–94.73%) and turkeys (100%, 3/3; 95% CI: 29.24–100%). One *C. jejuni* isolate from broilers (12.5%, 1/8; 95% CI: 0.32–52.65%) lacked all colonization genes. Three-gene profiles (*cdt*A-*cdt*B-*cdt*C) were more frequent in isolates from turkeys than broilers for both *C. jejuni* (100%, 3/3; 95% CI: 29.24–100% vs. 40%, 2/5; 95% CI: 5.27–85.34%) and *C. coli* (77.78%, 7/9; 95% CI: 39.99–97.19% vs. 30%, 3/10; 95% CI: 6.67–65.25%). Overall, ten distinct virulence profiles were identified in poultry.

Among *C. jejuni* and *C. coli* 62.5% (5/8) and 10.5% (2/19) carried all virulence genes (*cad*F-*rac*R-*dna*J-*cdt*A-*cdt*B-*cdt*C), respectively. There is no *Campylobacter coli* isolated from broiler harboured all 6-virulence gene together, while 40% (2/5) of *C. jejuni* isolated from broiler carried all genes. While in turkeys, 22.22% (2/9) of *C. coli* and 100% (3/3) of *C. jejuni* harbored all virulence associated genes, indicating that turkey *C. jejuni* isolates may represent the most virulent strains.

Only *rac*R was detected in *C. lari*.

### 3.4 Molecular analysis of *fla*A-RFLP results

**Figure 1:**
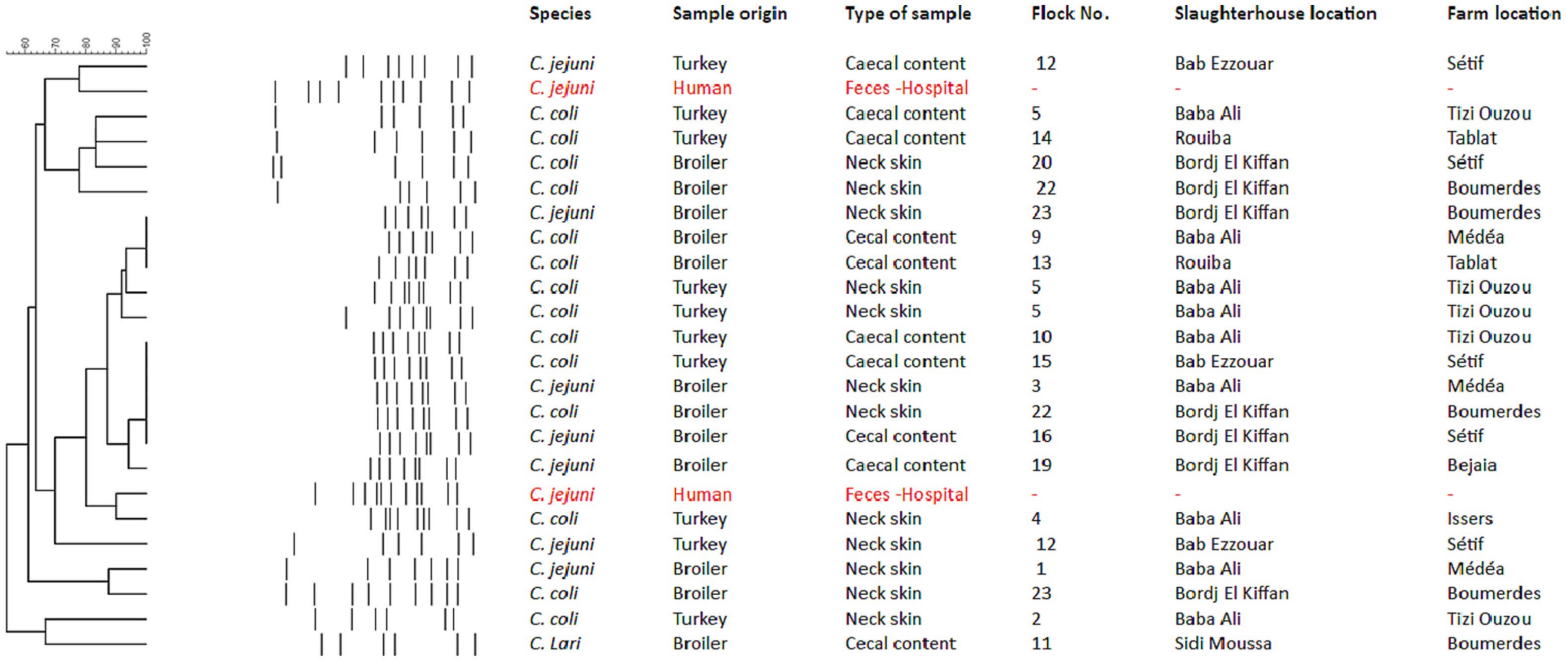
Dendrogram based on restriction profiles of 24 *Campylobacter* isolates were digested using *Sau3*AI and D*deI* restriction enzymes. *fla*A-RFLP cluster analysis was performed with the Dice correlation coefficient and the unweighted pair group mathematical average clustering algorithm of BioNumerics V. 7.1.

The PCR-RFLP analysis of *fla*A genes of the 22 *Campylobacter* spp. isolated from poultry revealed 16 genotypes when digested with *Sau3*AI and *Dde*I (Figure 1). All isolates gave identical results when experiments were repeated (data not shown). The two *C. jejuni* isolated from humans were not genotypically related to *Campylobacter* spp. isolated from poultry in this study.

## 4 Discussion

According to previous studies, variations in *Campylobacter* prevalence in poultry are influenced by multiple factors, including season, geographic location, detection methods, sample size and sample type (34, 35).

In this study, the overall prevalence of thermotolerant *Campylobacter* in the slaughterhouses was 21.05%. Despite a higher number of neck skin samples (*n* = 115) compared to cecal contents (*n* = 18), *Campylobacter* detection was higher in cecal contents (61.11%) than in neck skins (14.78%). This pattern is consistent with the fact that turkeys and broilers serve as primary reservoirs of thermotolerant *Campylobacter*, with higher bacterial loads in the intestinal tract than on neck skin in agreement with previous reports (36, 37).

The low detection rate may also be influenced by season, as most samples were collected during winter (12 batches) and autumn (8 batches), with only three batches in spring and none in summer. Peak contamination typically occurs in summer, decreasing thereafter (38). Additionally, *Campylobacter* are highly sensitive to desiccation; sampling from chilled carcasses may further reduce detection rates due to moisture loss and cold-induced inactivation during chilling and ventilation processes (39).

In poultry, *Campylobacter* prevalence in the intestinal tract is higher than that of *Salmonella* (40). Both broilers and turkeys are important hosts; colonization typically begins around 7 days of age, reaching near 100% within 14–28 days (41). Transmission within a flock is rapid, potentially infecting all birds within 72 h (24).

Three species were isolated in descending order of prevalence: *C. coli* (67.86%), *C. jejuni* (28.57%) and *C. lari* (3.57%). While some studies report *C. coli* as dominant in turkeys and broilers (42), most literature indicates *C. jejuni* predominance (61.9–73%), with *C. coli* ranging from 0–14.3% (43, 44).

Environmental stress responses differ between species. *Campylobacter jejuni* is generally more resilient to environmental stressors, whereas *C. coli* may survive better after transport and handling due to shifts in intestinal microflora (42, 45). *Campylobacter lari* is typically found in gulls but can cause severe gastrointestinal infections or bacteremia in humans (46).

Based on the available breakpoints reported for *Campylobacter* in both EUCAST and CLSI, the breakpoints were limited available only for fluoroquinolones, tetracyclines and macrolides. In this study, we tried to cover other clinically used antimicrobial agents used in the poultry farms in Algeria. For this reason, the resistance breakpoints for *Enterobacteriaceae* were used for Ampicillin (AM), Amoxicillin/Clavulanic Acid (AMC), Cefotaxime (CTX), Streptomycin (S), Gentamicin (GM), Kanamycin (K), Tobramycin (TM) and Chloramphenicol (C) as recommended by the Clinical and Laboratory Standards Institute and EUCAST (23, 47, 48).

The broth microdilution is considered the “gold standard” for determining minimum inhibitory concentrations (MICs). The disc diffusion is highly regarded for its simplicity, cost-effectiveness and flexibility. According to financial limit, disc diffusion (Kirby-Bauer) testing offers several practical advantages in routine clinical settings, small laboratories or when flexibility is required. Disc diffusion is significantly more cost-effective than broth microdilution, particularly for smaller laboratories or when testing a low number of samples (16, 49).

In this study, using the disc diffusion give flexibility for easily choose of antibiotic discs, allowing us to customize the panel to fit clinically relevant antimicrobial agents. Furthermore, the method is technically simple to perform, does not require specialized equipment, and is straightforward for laboratory technicians to interpret.

Nevertheless, several studies have demonstrated that the broth dilution method can be substituted by agar disk diffusion and the E-test, as the results obtained with these techniques show strong agreement with those of the dilution method (50–55). It should also be noted that the agar disk diffusion method was first standardized by the European Committee on Antimicrobial Susceptibility Testing (EUCAST) (16), and subsequently by the CLSI, which also recommends its use (56).

According to Yildirim *et al*. (2005), the agar disk diffusion test is a reliable, simple, and cost-effective method for determining the antibiotic susceptibility of *C. jejuni* and *C. coli* strains (54). The E-test, meanwhile, may also be reliable but requires a prior standardization step before it can be routinely used as a susceptibility testing method for *Campylobacter* strains (54, 55, 57).

High resistance rates were observed for nalidixic acid (96.43%), ciprofloxacin (92.86%) and tetracycline (85.71%), likely due to excessive use in poultry farms. Fluoroquinolone resistance is mainly chromosomal, associated with mutations in the *gyr*A gene QRDR region (Thr-86, Asp-90, Ala-70) and, to a lesser extent, by the CmeABC efflux pump (20, 23, 47, 58–60).

*Campylobacter jejuni* isolated from broilers, laying hens, and farm workers in Egypt demonstrated high rates of resistance to ampicillin (69.6%), ciprofloxacin (68.1%), erythromycin (66.7%), amoxicillin (65.2%), tetracycline (63.8%) and azithromycin (63.8%). In contrast, the lower rates of resistance to several other antibiotics ranged from 34.8 to 47.9% (61). While in Pakistan, the antimicrobial resistance rates of *C. jejuni* and *C. coli* in humans, animals and the environment were 89, 75, 70, 69, 54, 39 and 23% against tetracycline, ceftriaxone, cefotaxime, erythromycin, nalidixic acid, ciprofloxacin and gentamicin, respectively (62).

Tetracycline resistance is plasmid- or chromosome-mediated, primarily involving *tet*O and, rarely, *tet*M, which protect ribosomes from antibiotic binding (63, 64). The present study confirmed high prevalence of *tet*O among tetracycline-resistant isolates, particularly in *C. jejuni* (100%) and *C. coli* (88.24%). Some discrepancies between phenotypic and genotypic antimicrobial resistance may reflect primer sensitivity or the presence of additional resistance genes (65).

Resistance to *β*-lactams, including ampicillin (75%) and cefotaxime (50%), is mainly due to chromosomal β-lactamase production, with efflux pumps and low permeability contributing (66). Amoxicillin/clavulanic acid-maintained activity due to β-lactamase inhibition. Aminoglycoside resistance (kanamycin, streptomycin, tobramycin; 35.71–53.57%) is often plasmid-mediated, involving AAD, AAC, and APH enzymes (66). Macrolide resistance (erythromycin; 53.57%) is mainly chromosomal, involving 23S rRNA mutations, with efflux pumps and membrane alterations contributing (28, 67).

Chloramphenicol resistance was low (3.57%), reflecting restricted use, while the CmeABC efflux pump or plasmid-encoded CAT enzymes could explain the rare resistant isolates (66, 68). High-level resistance to ciprofloxacin, erythromycin and tetracycline may result from combined mechanisms including *gyr*A or 23S rRNA mutations, *tet*O and efflux pumps (58).

The ability of thermotolerant *Campylobacter* to colonize the intestine and cause infection in humans and animals is largely mediated by specific virulence genes (69, 70). In this study, the colonization genes *cad*F, *rac*R and *dna*J were the most frequently detected, confirming their major role in bacterial adhesion and survival in the host intestine (69, 71, 72). The *cad*F gene encodes a fibronectin-binding protein essential for bacterial adherence to intestinal epithelial cells (72). Similarly, *rac*R is a transcriptional regulator that modulates the expression of multiple colonization factors (71), while *dna*J is involved in stress response and contributes to bacterial survival in the gastrointestinal environment (69).

Regarding pathogenicity associated genes, *cdt*A, *cdt*B and *cdt*C, which encode the cytolethal distending toxin (CDT), were detected with variable frequencies depending on the species and the origin of the isolates (70). CDT is known to cause DNA damage in host cells, leading to cell cycle arrest and cell death (73, 74). The results of this study show that *C. jejuni* more frequently harbored the full set of *cdt* genes compared to *C. coli*, which may explain the higher potential virulence of this species in humans (70, 75).

The combination of colonization and pathogenicity associated genes suggests that certain strains may exhibit a potential complete virulence profile (*cad*F-*rac*R-*dna*J-*cdt*A-*cdt*B-*cdt*C), which might facilitate both intestinal colonization and the onset of severe gastrointestinal symptoms (70, 75). This observation is consistent with previous studies, which demonstrated that the presence of these genes together is associated with increased colonization and virulence potential (69, 70). Finally, our findings suggest that the prevalence of virulence genes may be influenced by the sample origin (broiler chicken *vs*. turkey), and that species such as *C. lari* possess a more limited set of virulence genes, which could explain their lower clinical incidence in humans (70, 76).

The genetic diversity amongst *Campylobacter* must be considered in epidemiological evaluations and microbial risk assessments of *Campylobacter* in poultry and poultry meat. Multiple genotypes can constitute the *Campylobacter* population within poultry flocks, suggesting different sources of exposure and/or genetic drifts within the *Campylobacter* population (77).

Molecular methods used for typing of *Campylobacter* spp., which are characterized by low complexity and high reproducibility, are needed to study the bacterial population structure. The use of *fla*A gene typing for epidemiological studies is controversial, due to the intra- and inter-genomic recombination within the flagellin genes that results in significant sequence heterogeneity (78). The discriminatory power of *fla*A-RFLP typing clearly depended upon the type of restriction enzyme used (14, 78, 79).

There are various methods used for typing *Campylobacter* as MLST, cgMLST and Whole Genome Sequencing which are considered the modern molecular tools for genotyping. The limitation in this study was based on the financial limit. According to previously published reports, it has been suggested that the sensitivity of the *fla*A gene locus to spontaneous genetic change is a limiting factor in its use in long-term epidemiological studies, but is suitable for the initial grouping of isolates in surveillance situations (80). The *fla*A gene of *Campylobacter* species serves as an epidemiological marker, as it shows extensive sequence heterogeneity (79). The *fla*A typing in *Campylobacter* is a commonly used, rapid and easy method for genotyping with an acceptable discriminatory power (81, 82). It has been shown that PCR-RFLP of *fla*A amplicons was suitable for discriminating *Campylobacter* isolates by generating DNA banding pattern. Different restriction enzymes can be used, and combining the enzyme patterns (composite analysis) has been shown to result in an increased degree of discrimination (14). Furthermore, the method is technically simple to perform, does not require specialized equipment, and is straightforward for laboratory technicians to interpret.

In this study, the genetic diversity among 22 *Campylobacter* spp. isolated from poultry and two *C. jejuni* isolated from human isolates was elucidated. Molecular biological typing was done using established *fla*A typing method. The present study demonstrated that poultry flocks can be simultaneously colonized with more than one *Campylobacter* genotype which in accordance with previous reports (14, 77). In contrast, other studies reported the detection of only one genotype per sampled flock (83, 84).

## 5 Conclusion

This study highlights the widespread presence of thermotolerant *Campylobacter* spp., particularly *C. jejuni* and *C. coli*, in poultry carcasses from slaughterhouses. The isolates exhibited high rates of antimicrobial resistance, including multidrug resistance and harbored multiple virulence genes associated with intestinal colonization and pathogenicity. These findings indicate that poultry products, even after refrigeration, may serve as reservoirs for pathogenic and antimicrobial-resistant *Campylobacter*, posing a significant public health risk.

The results also emphasize the need for improved hygiene practices, biosecurity measures, and targeted interventions along the poultry production and processing chain to limit bacterial contamination and the spread of resistance genes. Further research on the genetic mechanisms underlying resistance and potential virulence, as well as the development of standardized detection protocols, is essential to reduce the impact of *Campylobacter* contamination and protect human from infection.

## Data Availability

The raw data supporting the conclusions of this article will be made available by the authors, without undue reservation.
